# The Correlation of Il28B Genotype With Sustained Virologic Response In Romanian patients With Chronic Hepatitis C

**DOI:** 10.5812/kowsar.1735143x.4202

**Published:** 2011-12-20

**Authors:** Ioan Sporea, Alina Popescu, Manuela Curescu, Roxana Sirli, Isabel Dan, Adrian Goldis, Oana Gradinaru, Melania Ardelean, Mirela Danila, Simona Bota, Alexandra Deleanu

**Affiliations:** 1 Department of Gastroenterology and Hepatology, University of Medicine and pharmacy, Timisoara, Romania; 2 Department of Infectious Diseases, University of Medicine and pharmacy, Timisoara, Romania

**Keywords:** Genetic Variation, IL28B Protein, Human, Hepatitis C, Chronic

## Abstract

**Background:**

Multiple variables influencing the sustained virologic response (SVR) in chronic hepatitis C have been evaluated. One of them is genetic polymorphism near the IL28B gene.

**Objectives:**

The aim of this study was to evaluate the influence of IL28B genotypes on SVR rates in a group of patients with chronic hepatitis C from the western part of Romania.

**Patients and Methods:**

A retrospective study was performed in 107 consecutive patients, previously treated with standard-of-care medication for chronic hepatitis C, identified from the databases of 2 centers. Patient demographics, viral load before treatment and at 12, 24, and 72 weeks from the treatment start, and IL28B genotype were evaluated.

**Results:**

Among the 107 patents in the study group, 54 patients had SVR (50.5%), and 62 (57.9%) showed a complete early virologic response (cEVR). The SVR rates according to IL28B genotype were as follows: 73.1% in patients with genotype C/C, 40.9% in those with genotype C/T, and 57.1% in those with genotype T/T (i.e., 73.1% among patients with the C/C genotype vs. 43.7% among those with non-C/C genotypes; P = 0.0126). The cEVR rates were 80.8% in patients with the C/C genotype vs. 51.2% in those with non-C/C genotypes (P = 0.011).

**Conclusions:**

In our cohort of 107 Caucasian HCV patients, the SVR rate was 50.5% with standard-of-care treatment. The SVR rate was directly related to the IL28B genotype: 73.1% in the C/C genotype vs. 43.7% in non-C/C genotypes (P = 0.0126).

## 1. Background

Chronic C hepatitis is a global healthcare problem, affecting approximately 3% of the world population [1], and is an endemic disease in some areas [2]. The number of patients requiring treatment for this disease is increasing; therefore, the treatment costs, at the global level, are huge. Reference studies report sustained virologic response (SVR) rates ranging from 34% to 61% in patients infected with genotype 1 HCV, in connection with ethnicity, gender, age, severity of fibrosis, or viral load [3][4][5]. Multiple factors influencing SVR rates have been evaluated, to find the best candidates to achieve viral clearance. Recently, several papers reported that a genetic polymorphism near the IL28B gene, encoding interferon-lambda-3, is associated with an approximately 2-fold change in treatment response [6][7][8]. Several previous studies showed that SVR rates vary in patients according to their ethnicity, with lower rates in the Afro-American population followed by Caucasians, and with the best results occurring in Asian populations. These results can be possibly explained by genetic polymorphisms in the IL28B gene in different populations. In Romania, 99.7% of HCV patients are infected with genotype 1, the most difficult to treat [9]. A recently published epidemiological study showed that 3.23% of the Romanian population is chronically infected with HCV [10]. Thus, considerable financial effort is needed to treat this large number of patients; therefore, if very good predictors for SVR are found, different approaches should be used for these patients. New drugs for the treatment of chronic hepatitis C (telaprevir or boceprevir) are expected on the market in the next year, with better therapeutic results (compared to pegylated interferon [PegIFN] and ribavirin alone), but probably with high financial impact. New therapeutic strategies for the stratification of patients may therefore be needed in order to increase SVR rates with minimal cost increase.

## 3. Materials And Methods

We performed a retrospective study on 107 consecutive unrelated Caucasian patients previously treated for chronic hepatitis C with standard of care medication: PegIFN α-2a (180 µg/week) + ribavirin (1,000 mg/day for patients with body weight < 75 kg; 1,200 mg/day for patients with body weight > 75 kg) for 48 weeks; or PegIFN α-2b (1.5 µg/kg/week) + ribavirin (800 mg/day for patients with body weight < 65 kg; 1,000 mg/day for patients with body weight 65-85 kg; 1,200 mg/day for patients with body weight > 85 kg) for 48 weeks. Clinically significant dose reductions of either PegIFN or ribavirin were not needed in any of the patients. Patients who received treatment starting from January 2009 and who finished the treatment regimen before 31st of May 2010 were identified from the databases of two centers. We contacted all patients who underwent treatment in this period and included all who accepted to participate in the study. Each patient's data were collected in a specially designed study file that included patient demographics, body mass index (BMI), severity of fibrosis evaluated on liver biopsy by the Metavir score (available in 102/107 patients), viral load before treatment, at 12 weeks of treatment (to evaluate the early virologic response, EVR), at 24 weeks of treatment (in patients in which only partial EVR was achieved), and at 72 weeks from the start of treatment (to evaluate the SVR). EVR was defined as undetectable viral load or a decrease in viral load by at least two log10 after 12 weeks of treatment, compared with baseline. Complete EVR (cEVR) was defined as undetectable viral load after 12 weeks of treatment. Partial EVR (pEVR) was defined as a decrease in viral load by at least two log10 compared to baseline after 12 weeks of treatment, but not aviremic. SVR was defined as undetectable viral load 24 weeks after the end of treatment. Blood samples were collected from each patient for IL28B genotyping, which was performed in the same unit, by real-time PCR (in-house method). Blood specimens were genotyped for IL-28B rsC12979860T on a LightCycler 1.5 Thermocycler (Roche, Mannheim, Germany). DNA was isolated from EDTA-whole blood using a fully automated purification device (GenoXtract) and the appropriate blood extraction cartridges (Hain Lifescience, Nehren, Germany). Following PCR amplification, the IL28B dimorphism was identified with a 6-carboxy fluorescein (FAM)-labeled HyBeacon [11] probe, which creates melting peaks at defined temperatures that are characteristic to specific DNA structures. Quantification of the HCV-RNA was done using the Cobas AmpliPrep/ Cobas TaqMan HCV [12] test (Roche) according to the recommendations of the manufacturer. Because a previous study performed in Romania showed that 99.7% of Romanian HCV patients are infected with genotype 1 [9], we decided not to perform HCV genotyping in our study group (in order to lower the costs). All the patients consented to participate to the study, which was approved by the local Ethical Committee. The data we obtained from our patients were collected in a Microsoft Excel file. Statistical analyses were performed using the Microsoft Excel 2007 and GraphPad Prism 5 programs. For the statistical study of quantitative variables, the mean and standard deviation were calculated. Unpaired t-test and one-way ANOVA test were used to compare means. Chi square test and Fisher's exact test were used to compare proportions.

## 4. Results

The study group included 107 patients, 63 (58.9%) women and 44 (41.1%) men, with a mean age of 53.4 ± 9.6 years. All patients were Caucasians. The mean BMI of the patients from the study group was 27.3 ± 3.9 kg/m2; 32.7% of the patients were of normal weight, 43.9% were overweight, and 23.4% were obese. The baseline viral load was < 600,000 IU/mL in 31.8% of patients, and the mean liver fibrosis (Metavir) score of the same group was 2.4 ± 0.6 (Table 1). From the study group, 54 patients achieved SVR (50.5%), while 53 (49.4%) did not (non-responders and relapsers). The frequencies of IL28B genotypes in the study group were as follows: 26 (24.3%) patients were of genotype C/C, 67 (62.6%) were of genotype C/T, and 14 (13.1%) were of genotype T/T. Table 2 summarizes patient characteristics in each IL28B genotype subgroup: mean age, gender distribution, mean BMI, mean viral load at baseline, number of patients with baseline viral load < 600,000 IU/mL, and mean liver fibrosis. The mean baseline viral load in each IL28B genotype subgroup was as follows: 2,250,100.5 IU/mL in the C/C group, 2,309,536.5 IU/mL in the C/T group, and 1,017,544.2 IU/mL in the T/T group. The baseline viral load in the T/T group was significantly lower than that in the C/C group (P = 0.0147) and the C/T group (P = 0.0084). The mean baseline viral loads in the C/C and C/T groups were not significantly different (P = 0.65). Further, the proportion of patients with a low baseline viral load (< 600.000 IU/mL) was highest in the T/T group (64.3%) compared to the C/C (19.2%) and C/T (28.4%) groups. There were no statistically significant differences between the three IL28B subgroups regarding the following parameters: mean fibrosis severity evaluated by the Metavir score (P = 0.8162 by one-way ANOVA test), mean BMI (P = 0.661 by one-way ANOVA test), mean age (P = 0.2388 by one-way ANOVA test), and the ratio of women to men (P = 0.358 by Chi-square test). The response rates in our study group stratified according to the IL28B genotype are presented in Table 3 (one of the 107 patients was noncompliant to treatment, despite the fact that he had a cEVR, and was excluded from the analysis). The occurrence of a complete early virologic response (cEVR) was significantly higher in the C/C genotype group (80.8%; 21/26 patients) than in the non-C/C genotypes (C/T + T/T; 51.2%; 41/80 patients; P = 0.011 by Fisher's exact test). On the other hand, SVR was achieved in 73.1% (19/26) of the C/C patients vs. 43.7% (35/80) of the non-C/C patients (P = 0.0126 by Fisher's exact test).

**Table 1:** Patient Characteristics in the Study Group

	**Value**
Patients, No.	107
Gender, No. (%)	
Women	63 (58.9)
Men	44 (41.1)
Age, y, mean ± SD a	53.4 ± 9.6
BMI a, kg/m^ 2^, mean ± SD	27.3 ± 3.9 b
Normal weight c, No. (%)	35 (32.7)
Overweight d, No. (%)	47 (43.9)
Obese e, No. (%)	25 (23.4)
Baseline viral load, IU/mL	
< 600,000	34 (31.8)
≥ 600,000	73 (68.2)
Liver fibrosis (Metavir) score, mean ± SD	2.4 ± 0.6
Distribution of liver fibrosis f, No. (%)	
F1 a	5 (4.9)
F2	49 (48)
F3	42 (41.2)
F4	6 (5.9)

^a^ Abbreviations: BMI, body mass index; F, fibrosis; SD, standard deviation

^b^ range 20.5.39.1 kg/m 2

^c^ < 25 kg/m 2

^d^ 25.29.9 kg/m 2

^e^ ≥ 30 kg/m 2

^f^ 102 patients with liver biopsy, evaluated according to the Metavir score

**Table 2:** Patient Characteristics in Each IL28B Genotype Subgroup

	**C/C Genotype**	**C/T Genotype**	**T/T Genotype**
Age, y, mean ± SD	54.2 ± 7.4	53.9 ± 10.1	49.7 ± 10.3
Patients, No.	26	67	14
Female, No. (%)	18 (69.2)	36 (53.7)	9 (64.3)
BMI a, kg/m ^2^, mean ± SD	26.6 ± 2.9	27.7 ± 4.2	26.9 ± 4.1
Viral load at baseline, IU/mL, mean ± SD a	2,250,100.5 ± 1,743,166	2,309,536.5 ± 2,420,368	1,017,544.2 ± 1,462,900.7
Patients with baseline viral load < 600,000 IU/mL, No. (%)	5 (19.2)	19 (28.4)	9 (64.3)
Liver fibrosis b, mean ± SD	2.4 ± 0.6 c	2.5 ± 0.7 d	2.3 ± 0.5 e

^a^ Abbreviations: BMI, body mass index; SD, standard deviation

^b^ in cases with liver biopsy – Metavir score

^c^ 26 patients

^d^ 63 patients

^e^ 13 patients

**Table 3:** Relationship Between IL28B Genotype and Response to Treatment Rates (cEVR, EVR and SVR) in the Study Group (106 patients)

	**C/C Genotype **	**C/T Genotype **	**T/T Genotype **
Patients, No.	26	66	14
SVR a, No. (%)	19 (73.1)	27 (40.9)	8 (57.1)
Non-SVR, No. (%)	7 (26.9)	39 (59.1)	6 (42.9)
cEVR a, No. (%)	21 (80.8)	34 (50.7)	7 (50)
EVR a (cEVR + pEVR a), No. (%)	26 (100)	60 (89.5)	12 (85.7)

^a^ Abbreviations: ceVR, complete early virologic response; eVR, early virologic response; peVR, partial early virologic response; SVR, sustained virologic response

## 5. Discussion

Several papers have reported that genetic polymorphisms near the IL28B gene, encoding interferon-lambda-3, influence the response to treatment in chronic hepatitis C [6][7][8]. In a study performed on 871 patients with Caucasian ancestry [13], the C/C genotype was found in 39%, C/T in 49%, and T/T in 12% of the study group. Consistent with these findings, the majority of the patients in the present study were of genotype C/T (62.8%), followed by 24.5% with genotype C/C, and a small proportion (12.7%) with genotype TT. Studies originating from different parts of Asia indicate that a large proportion of the Asian population is of IL28B genotype C/C; this explains the high SVR rates in patients with chronic hepatitis C in this area [14][15]. The most important aspect of IL28B genotyping is its relationship with SVR rates following standard-of-care treatment (PegIFN plus ribavirin). Two years ago, a paper published in Nature by Ge et al. [6] showed that in patients of European ancestry, the C/C genotype is associated with a 2-fold (95% confidence interval 1.8-2.3) greater rate of SVR than in the T/T genotype. Another study [16] showed that in patients with the C/C genotype, the SVR was 69%, compared to 33% in those with the C/T genotype and 27% in those with the T/T genotype. In the same study, important differences were observed regarding several other parameters: the rapid virologic response (RVR; 28%, 5%, and 5% in patients with the C/C, C/T, and T/T genotypes, respectively); EVR (97%, 72%, and 68% in patients with the C/C, C/T, and T/T genotypes, respectively); the end-of-treatment response (EOT; 92%, 56%, and 51% in patients with the C/C, C/T, and T/T genotypes, respectively); relapse rates (14%, 27%, and 31% in patients with the C/C, C/T, and T/T genotypes, respectively). Another group tested if the IL28B genotype could be used to tailor treatment duration according to baseline viral load and virologic response during treatment in genotype 1 HCV patients [17]. In this multicenter German study, 398 treatment-naive HCV genotype-1 patients were enrolled and treated with PegIFN and ribavirin. Overall SVR rates of 55% and 48% were obtained in patients treated with individualized durations and 48 weeks standard duration, respectively. The IL28B genotype was available for 305 of the 398 patients who completed therapy. The SVR rate in patients who completed the therapy was 65%. Of the non-responders, 96% (65/68) were of IL28B genotypes C/T and T/T. SVR was achieved in 85%, 58%, and 46% of patients with C/C, C/T, and T/T IL28B genotypes, respectively. However, the overall relapse rates were similar for C/C, C/T, and T/T IL28B genotypes (13%, 19%, and 21%, respectively). A study by Stättermayer and co-workers [18] evaluated the role of IL28B genotypes on the EVR in 682 treatment-naïve patients, including 372 patients infected with HCV genotype (GT) 1, 208 patients with GT 2/3, and 102 patients with GT 4. Patients were treated with 180 μg PegIFN α-2a, and 400 or 800 mg (GT 2/3, depending on the protocol) or 1000-1200 mg (GT 1/4) ribavirin per day, the duration of treatment being 24 (GT 2/3) or 24-72 weeks (GT 1/4). This study showed that the viral load decrease 24 hours after the first dose of interferon in patients of GT 1/4 was greater in carriers of the C/C than of the T/T allele, and that patients with the C/C allele also had higher rates of RVR (GT 1, 38.3% vs. 11.6%; GT 4, 76.5% vs. 23.5%; both P < 0.001) and SVR (GT 1, 79.1% vs. 43.2%; GT 4, 85.3% vs. 44.1%; both P < 0.001). The conclusion of this study was that an early virologic response to PegIFN and ribavirin is more likely among carriers of the C/C IL28B polymorphism, which might underlie their high rate of SVR, and that IL28B genotype determination as well as the presence of an RVR might be used in future studies of patients with hepatitis C virus genotypes 1 or 4. Studies regarding the relationship between the IL28B genotype and the results of treatment in patients infected with HCV GT 2 and 3 showed that the influence of the IL28B genotype on the SVR in these patients is less important [18][19][20]. Currently, there are several published papers regarding the relationship between the IL28B genotype and SVR (also RVR or EVR) in Caucasian patients infected with HCV genotype 1. In the German study previously mentioned, the overall SVR after 48 weeks, with standard duration of treatment, was 48% [17]. In our study in the western part of Romania, with the same standard 48-week treatment duration, the SVR was 50.5 %, similar to the German study. Additionally, in a previously published study from our group that included 582 HCV genotype 1 patients from 2 Romanian centers, in an intention-to-treat analysis, the SVR rate was 51.9% [5]. The most important aspect of IL28B genotyping is the fact that we can predict the chance of SVR (and, according to some studies, the chance of RVR and EVR), based on the IL28B genotype of the patient. A study by Thompson et al. [16] showed that patients with the C/C genotype achieved an SVR rate of 69%, as compared to 33% and 27% in those with the C/T and T/T genotypes, respectively. In a study by Sarrazin et al. [17], SVR was achieved in 85%, 58%, and 46% of patients with the C/C, C/T and T/T IL28B genotypes, respectively. In the group of patients from the Stättermayer study [18], the SVR rate was 79.1% in the C/C genotype vs. 43.2% in patients with T/T genotype. In our group, similar results were obtained: a 73.1% SVR rate in patients of genotype C/C, 40.9% in patients of genotype C/T, and 57.1% in patients of genotype T/T (or 73.1% in C/C vs. 43.7% in non-C/C patients; P = 0.0126). On the other hand, in our study, the baseline viral load in the T/T group (1,017,544.2 IU/mL) was significantly lower than in the C/C (P = 0.0147) and C/T (P = 0.0084) groups, and the proportion of patients with a low baseline viral load (< 600.000 IU/mL) was the highest in the T/T group (64.3%), compared to the C/C (19.2%) and C/T (28.4%) groups. These differences, and the small number of patients in the T/T group, may explain the high SVR in this group (SVR = 57.1%). Lagging et al. [21] carried out a study involving 253 Caucasian patients, in whom they correlated the occurrence of variants at 3 such SNPs (rs12979860, rs12980275, and rs8099917) with pretreatment plasma IP-10 and HCV RNA levels throughout therapy. They demonstrated that in genotype 1-infected patients, baseline IP-10 and a C genotype at rs12979860 independently predicted the first phase of viral decline and RVR, which in turn independently predicted SVR. Thus, among genotype 1-infected patients, homozygous carriers of the 3 favorable IL28B alleles had significantly more pronounced first phase viral decline than did patients carrying the risk alleles (mean, 2.0, 0.9, and 0.6 log10 IU/mL for rs12979860 CC, CT, and TT, 1.8, 0.9, and 0.7 log10 IU/mL for rs12980275 AA, AG, and TT, respectively, and 1.4, 0.8, and 0.6 log10 IU/mL for rs8099917 TT, TG, and GG respectively; P = 0.0001 for all 3 SNPs; Kruskal-Wallis test). Furthermore, among HCV genotype 1-infected carriers of the favorable C, A, or T alleles, IP-10 levels below 150 pg/mL significantly predicted higher rates of RVR (62%, 53%, and 39%) and SVR (85%, 76%, and 75% respectively). On the other hand, patients carrying favorable SNP genotypes had a higher baseline viral load than those carrying unfavorable variants. The authors concluded that concomitant assessment of pretreatment IP-10 levels and IL28B-related SNPs augments the prediction of RVR and the final therapeutic outcome. Thus, from the perspective of triple therapy for HCV chronic infection that is expected to enter soon in current clinical practice, testing for the IL28B genotype of the patient seems to be reasonable, in order to make a decision regarding the treatment. In the C/C genotype, the high SVR rates possibly justify treating these patients with double therapy (PegIFN + ribavirin) only, and for the C/T and T/T genotypes, triple therapy seems to be the best option.

In our cohort of 107 Caucasian patients with chronic hepatitis due to HCV, the SVR rate was 50.5% with standard-of-care treatment (PegIFN and ribavirin for 48 weeks). The SVR rate was directly related to the IL28B genotype: 73.1% in the C/C IL28B genotype vs. 43.7% in non-C/C patients (P = 0.0126). Further treatment strategies for chronic hepatitis C can be designed based on the IL28B genotype, in order to increase the SVR rate.
